# Immunosenescence: the potential role of myeloid-derived suppressor cells (MDSC) in age-related immune deficiency

**DOI:** 10.1007/s00018-019-03048-x

**Published:** 2019-02-20

**Authors:** Antero Salminen, Kai Kaarniranta, Anu Kauppinen

**Affiliations:** 1 Department of Neurology, Institute of Clinical Medicine, University of Eastern Finland, P.O. Box 1627, 70211 Kuopio, Finland; 2 Department of Ophthalmology, Institute of Clinical Medicine, University of Eastern Finland, P.O. Box 1627, 70211 Kuopio, Finland; 3 Department of Ophthalmology, Kuopio University Hospital, KYS, P.O. Box 100, 70029 Kuopio, Finland; 4 School of Pharmacy, Faculty of Health Sciences, University of Eastern Finland, P.O. Box 1627, 70211 Kuopio, Finland

**Keywords:** Aging, Cellular senescence, Immunotherapy, Myelopoiesis, Rejuvenation, Trained immunity

## Abstract

The aging process is associated with chronic low-grade inflammation in both humans and rodents, commonly called inflammaging. At the same time, there is a gradual decline in the functional capacity of adaptive and innate immune systems, i.e., immunosenescence, a process not only linked to the aging process, but also encountered in several pathological conditions involving chronic inflammation. The hallmarks of immunosenescence include a decline in the numbers of naïve CD4^+^ and CD8^+^ T cells, an imbalance in the T cell subsets, and a decrease in T cell receptor (TCR) repertoire and signaling. Correspondingly, there is a decline in B cell lymphopoiesis and a reduction in antibody production. The age-related changes are not as profound in innate immunity as they are in adaptive immunity. However, there are distinct functional deficiencies in dendritic cells, natural killer cells, and monocytes/macrophages with aging. Interestingly, the immunosuppression induced by myeloid-derived suppressor cells (MDSC) in diverse inflammatory conditions also targets mainly the T and B cell compartments, i.e., inducing very similar alterations to those present in immunosenescence. Here, we will compare the immune profiles induced by immunosenescence and the MDSC-driven immunosuppression. Given that the appearance of MDSCs significantly increases with aging and MDSCs are the enhancers of other immunosuppressive cells, e.g., regulatory T cells (Tregs) and B cells (Bregs), it seems likely that MDSCs might remodel the immune system, thus preventing excessive inflammation with aging. We propose that MDSCs are potent inducers of immunosenescence.

## Introduction

The aging process in humans is associated with a gradual decline in the functional capacity of adaptive and innate immune systems [1–5]. This age-related immune deficiency has been called immunosenescence. Clinically, immunosenescence reduces vaccination efficiency and impairs anticancer immunity, thus increasing the susceptibility to infections and the prevalence of cancers with aging. Immune deficiencies, similar to those encountered in immunosenescence, also appear in many diseases involving chronic inflammation, e.g., sepsis and autoimmune diseases. It is known that chronic low-grade inflammation, called inflammaging, down-regulates the immune responses of both the adaptive and innate immune system in humans and mice [3]. Not only does aging modulate the phenotypes and functions of immune cells, but it also affects their development and maturation in the bone marrow and spleen. Currently, it is still unclear whether immunosenescence is a significant defense mechanism against age-related chronic inflammation or a detrimental consequence of the chronic low-level inflammatory condition associated with aging. However, it is known that the aging process induces an active remodeling of the immune system rather than causing irreversible cellular senescence such as that occurring in non-immune cells [6, 7].

**Fig. 1:**
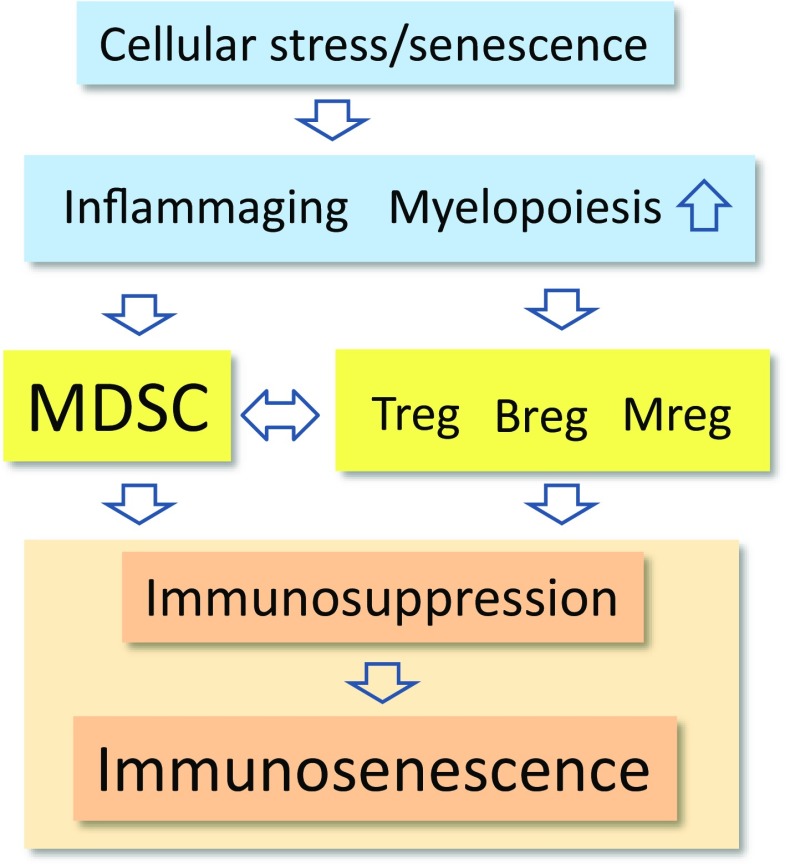
A schematic representation of MDSC-driven immunosenescence. The age-related cellular stress and senescence induce a condition termed inflammaging, which is associated with increased myelopoiesis. A mild inflammatory profile stimulates the production of MDSCs and other immunosuppressive cells, e.g., Tregs, Bregs, and Mregs. The cooperation between the components of this immunosuppressive network creates an immune-suppressive microenvironment, which after the remodeling of the immune system generates immunosenescence

There is convincing evidence that chronic inflammation induces immunosuppression which inhibits both adaptive and innate immunity in different human disorders [8, 9]. Inflammatory factors promote the recruitment of immunosuppressive cells into inflamed tissues, where they suppress persistent inflammation and restore homeostasis in inflamed tissues. Myeloid-derived suppressor cells (MDSC) are specialized immunosuppressors which can control the functions of other immune cells, thus preventing excessive inflammatory responses [10, 11] (Fig. 1). For instance, Bunt et al. [12] demonstrated that chronic inflammation increased the accumulation of MDSCs into mouse mammary carcinoma; this induced immunosuppression, allowing the tumor cells to undergo immune escape. The inhibition of inflammation prevented the recruitment of MDSCs into tumors, a process that prevented the immunosuppression and subsequently blocked tumor growth. It is not only cancer-related inflammation which recruits MDSCs since these cells accumulate in many inflamed, non-neoplastic tissues and consequently suppress the functions of T cells and myeloid cells [13]. We will compare the immune profiles of immunosenescence and MDSC-driven immunosuppression. This comparison clearly highlights how immunosenescence might be driven by MDSCs which modulate the immunosuppressive network to the form encountered in both inflammaging and many inflammatory diseases. Bueno et al. [14] have also speculated that MDSCs could be involved in the generation of immunosenescence.

### Immunosenescence

**Fig. 2:**
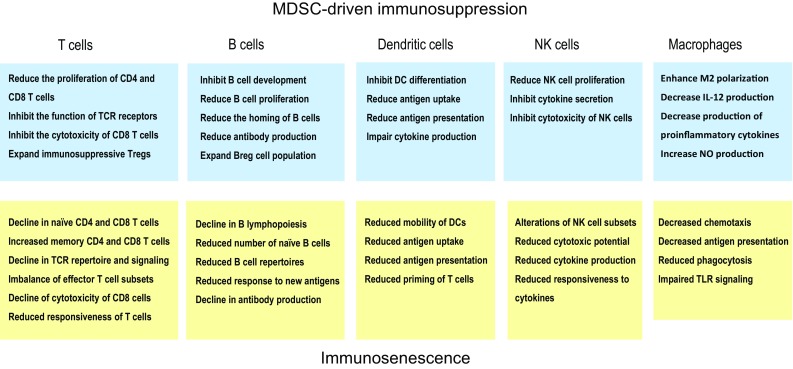
The comparison of immune cell phenotypes induced by MDSC-driven immunosuppression (upper panel) and age-related immunosenescence (lower panel)

There is a substantial literature indicating that a distinct functional decline occurs in the human immune system with aging, although all age-related alterations are not ubiquitous since many of these modifications seem to be context dependent [1, 3, 5, 15]. Briefly, an involution of the thymus and a marked decline in the numbers of naïve CD4^+^ and CD8^+^ T cells are the common biomarkers of immunosenescence in both humans and mice. Correspondingly, there is an expansion of memory CD4^+^ and CD8^+^ T cells, which might be a reflection of a persistent antigen load, e.g., induced by cytomegalovirus (CMV) infections with aging in humans [16, 17]. In addition, there are distinct deficiencies in the B cell compartment with aging which disturb the maintenance of humoral immunity, e.g., the decline in antibody production [18, 19]. Overall, the effects of aging are more profound on adaptive immunity than on innate immunity, although there exist also some functional deficiencies in dendritic cells [20], monocytes/macrophages [21], and natural killer cells [22] (Fig. 2). Interestingly, age-related immunosenescence seems to be an evolutionarily conserved phenomenon, e.g., in insects, birds, and mammals [23–25]. We will examine more thoroughly the specific changes in immunosenescence and compare their characteristics to those induced by MDSCs.

Currently, there is still a debate about whether immunosenescence evokes inflammaging or whether it is inflammaging which reprograms the immune system [7, 15]. Given that inflammation is a consequence rather than the original perpetrator of the aging process, it seems reasonable to argue that chronic inflammation could adapt the immune system to cope with the aging microenvironment. The inhibition of T cells and some components of innate immunity might protect tissues from excessive injuries in conditions where persistent insults cannot be removed and the resolution of inflammation is impaired. Moreover, immunosenescence is associated not only with the aging process, but is also present in different pathological conditions involving chronic inflammation. For instance, inflammation has a crucial role in tumorigenesis where there is a significant increase in the biomarkers of immunosenescence, e.g., in breast cancer [26], multiple myeloma [27], glioma [28], and lung cancer [29]. In addition, it has been recognized that immunosenescence has a crucial role in the pathology of rheumatoid arthritis [30] and cardiovascular diseases [31]. It is known that the impaired resolution of acute inflammation induces chronic inflammation involving immune suppression, which provokes detrimental effects in host tissues [8]. It seems that persistent inflammatory conditions cause an adaptive response in the immune system by inducing a state of immunosuppression, similar to immunosenescence, not only in the aging process but also in inflammatory diseases.

### MDSC-induced immunosuppression

MDSCs are a heterogeneous group of immunosuppressive myeloid cells which develop from common myeloid progenitor cells during the myelopoietic process [10, 32, 33]. Generally, we can separate these cells into human/mouse monocytic and granulocytic MDSC subsets which possess distinct phenotypes in their cell-surface markers as well as displaying some differences in their context-dependent immunosuppressive functions [33–35]. MDSCs are the major immune-suppressive cells in the body and thus they are involved in the host defense against inflammatory insults induced by either endogenous damages or by environmental insults, such as viral and bacterial infections [10, 36, 37]. Inflammatory factors, e.g., colony-stimulating factors (CSF), chemokines, and some cytokines can provoke emergency myelopoiesis in the bone marrow by stimulating the expansion and release of MDSCs [32, 38]. Chronic inflammation can also stimulate extramedullary myelopoiesis and trigger the generation of MDSCs, e.g., in the spleen and peripheral lymphoid organs. Several chemokines, e.g., CCL2, CXCL2, and IL-8, are able to induce the recruitment of MDSCs into inflamed tissues where they inhibit acute inflammatory responses allowing the resolution of inflammation [36, 37, 39, 40]. In inflamed tissues, many cytokines, e.g., IL-1β, IL-6, and TNF-α, as well as many inflammatory alarmins, such as HMGB1, S100 factors, and PGE2, can activate the immunosuppressive armament of MDSCs [36, 41–43]. The JAK-STAT and the NF-κB signaling pathways are the two major mechanisms involved in inducing the immunosuppressive potential of MDSCs [44]. Moreover, hypoxia/HIF-1α is a potent enhancer of MDSC-mediated immunosuppression [45].

MDSCs possess a powerful array of immune-suppressive mechanisms [10, 11, 46]. For instance, MDSCs secrete IL-10 and TGF-β cytokines which are potent anti-inflammatory and immunosuppressive factors. IL-10 and TGF-β are the major regulators of many of the functions performed by myeloid and lymphoid cells [47, 48]. For instance, IL-10 inhibits NF-κB signaling, a crucial inducer of pro-inflammatory reactions as well as stimulating STAT3 signaling, a key factor in the activation of MDSCs and immunosuppressive regulatory T cells (Tregs) [49–51]. Moreover, IL-10 can suppress the antigen presentation of dendritic cells and macrophages, and evoke macrophage M2 polarization [52, 53]. Similarly, TGF-β cytokines are potent human immunoregulators since TGF-β signaling can (1) convert naïve CD4^+^ cells into Tregs [54], (2) prevent T cell proliferation and the differentiation of Th1 and Th2 cells [47], (3) inhibit B cell responsiveness [55], (4) suppress human dendritic cell function [56], and (5) promote alternative M2 polarization in human macrophages [57]. In addition, TGF-β exerts degenerative bystander effects in non-immune cells, e.g., it can increase tissue fibrosis [58] and trigger cellular senescence in human fibroblasts [59]. However, MDSCs are not the only immune cells which secrete IL-10 and TGF-β cytokines, since other immunosuppressive cells also utilize the same mechanism to maintain immune suppression.

MDSCs alter the tissue microenvironment by actively generating reactive oxygen species (ROS) [33, 34, 60]. Corzo et al. [60] revealed that the STAT3 transcription factor, the major regulator of MDSC activation, induced the expression of NADPH oxidase (NOX2) which stimulated the production of ROS compounds in different mouse and human tumor models. MDSCs also generate nitric oxide (NO) by inducing the expression of inducible nitric oxide synthase (iNOS) [61]. Nagaraj et al. [62] demonstrated that peroxynitrite (ONOO^−^) nitrated the tyrosine residues in the T cell receptor (TCR) in mouse CD8^+^ T cells, thus preventing the antigen-specific stimulation of CD8^+^ T cells. It seems that the production of ROS is dependent on the insult and the subtype of MDSCs activated, e.g., a bacterial insult induced ROS generation in mouse granulocytic MDSCs, whereas monocytic MDSCs produced NO [34]. MDSCs are very resistant to ROS compounds; this is thought to be the reason why these cells can survive in inflammatory milieu in conditions of oxidative stress [63, 64]. Beury et al. [63] demonstrated that the increased expression of nuclear factor E2-related factor 2 (Nrf2), a powerful survival factor of cells in oxidative stress, enhanced the survival of MDSCs in tumors by decreasing the level of intracellular ROS and the rate of apoptosis in infiltrated MDSCs. Recently, Ohl et al. [64] revealed that the constitutive activation of Nrf2 in mouse myeloid cells increased the proliferation capacity of MDSCs, which induced the expansion of MDSCs and developed splenomegaly attributable to the accumulation of MDSCs into the spleen. They also reported that the increased expression of Nrf2 affected the metabolism of MDSCs by enhancing the expression of several genes involved in glycolytic energy metabolism. Oxidant species produced by MDSCs not only suppress the activity of immune cells, but also exert robust unspecific responses which affect both immune and non-immune cells, enhancing immunosenescence in inflamed tissues [65, 66]. In addition to ROS generation, the activation of MDSCs induces the expression of arginase 1 (ARG1) and indoleamine 2,3-dioxygenase (IDO) which catabolize arginine and tryptophan amino acids, respectively [67, 68]. A consequence of this enzymatic activation is that there is a shortage of these amino acids which inhibits protein synthesis, thus preventing the proliferation of T cells and other pro-inflammatory cells in inflamed tissues.

MDSCs can also induce immunosuppression of T cells via the cellular contacts mediated by immune checkpoint proteins [69, 70]. There is an abundant literature on the inhibitory checkpoint receptors and their inhibitors, since these membrane receptors are promising targets in cancer therapy. Recently, it was revealed that activated MDSCs expressed programmed death-ligand 1 (PD-L1) receptor protein which can bind to the PD-1 receptor of T cells and thus suppress their function [71, 72]. Many other human immune cells, e.g., B cells, dendritic cells, monocytes, and mast cells, express the proteins of the immunosuppressive PD-1/PD-L1 system [73]. Lei et al. [74] demonstrated that MDSCs acted through the PD-1/PD-L1 pathway to impair the ability of murine alveolar macrophages to respond to a pneumonia infection. Tregs and Bregs also utilize the PD-1/PD-L1 system to induce T cell immunosuppression [75, 76]. The PD-1/PD-L1 system is a potent source of immunosuppression in infections and tumors, but its role in age-related immunosenescence needs to be clarified.

### Immunosuppressive cooperation between MDSCs, regulatory T and B cells, and macrophages

The immunosuppressive armament not only contains MDSCs, but also regulatory T cells (Treg), regulatory B cells (Breg), and regulatory macrophages (Mreg) which are also called M2c macrophages [77–79] (Fig. 1). There is a significant cross talk between these cell populations in an attempt to induce and maintain an immunosuppressive microenvironment in conditions of chronic inflammation, e.g., present in tumors and many inflammatory diseases. In general, IL-10 and TGF-β cytokines have a crucial role in this kind of communication within this immunosuppressive network. For instance, MDSCs can induce the differentiation of Tregs as well as enhance the expansion of Treg and Breg populations [80–83]. Consequently, activated Tregs can inhibit the functions of T cells, e.g., they suppress the proliferation of naïve/effector T cells [84]. Correspondingly, the stimulation of Bregs can (1) trigger the production of anti-inflammatory IL-10 and TGF-β cytokines, (2) inhibit the immune reactions mediated by Th1 cells, and (3) prevent autoimmune diseases [85]. In addition, one distinct subset of Bregs can convert resting CD4^+^ T cells into Tregs in mouse tumors [86]. Tregs and MDSCs can also establish a positive feedback loop, since Tregs stimulate the expansion and immunosuppressive activities of MDSCs [87]. On the other hand, Sinha et al. [88] reported that the activated T cells were able to induce the apoptosis of MDSCs in mice. MDSCs are known to express the death receptor Fas, whereas activated T cells secrete the Fas ligand (FasL). Sinha et al. [88] demonstrated that the Fas–FasL system controlled the numbers of MDSCs in circulation in the context of cancer and metastasis.

Myeloid-derived cells including MDSCs, monocytes, macrophages/microglia, dendritic cells, and natural killer (NK) cells reveal plastic phenotypes. For instance, macrophages can become polarized toward proinflammatory M1 and anti-inflammatory M2 properties in a context-dependent manner [89]. The polarization of macrophages is not fixed in vivo, but there is remarkable plasticity in the properties of the M1 and M2 subpopulations. Microenvironmental conditions control the polarization of macrophages in tissues. In inflammatory conditions, circulating monocytes will be recruited into inflamed tissues, where they differentiate into the M1-type of macrophages [90]. Human monocytic MDSCs can also be converted into inflammatory macrophages [91]. In particular, the exposure of proinflammatory TNF-α enhanced the maturation of human MDSCs into macrophages. Correspondingly, the interaction of MDSCs and macrophages potentiated the immune-suppressive capacities of both cell populations, e.g., in the tumor microenvironment [78, 92]. This bidirectional cross talk, both cell contact dependent and contact independent, robustly increased IL-10 production which consequently activated immunosuppressive Tregs and stimulated Th2 responses, whereas antigen presentation decreased, impairing the cytotoxicity of CD8^+^ and NK cells [78, 93]. In tumors, infiltrating monocytic MDSCs can be differentiated into the immunosuppressive M2 macrophages, commonly called tumor-associated macrophages (TAM) [94]. TAMs possess specific immune properties, probably educated by cancer cells, and thus they constitute the type M2d macrophage phenotype. It is the cooperation between Tregs and macrophages which also augments the immunosuppression in inflamed tissues. For instance, Tiemessen et al. [95] observed that human Tregs were able to polarize circulating monocytes/macrophages into the immunosuppressive M2 subtype. More recently, Riquelme et al. [96] demonstrated that the human Mregs were able to convert CD4^+^ T cells into FoxP3^+^ expressing, IL-10-secreting Tregs, which then suppressed T cell immunity and inhibited dendritic cell maturation. In addition, Lu et al. [97] reported that mouse Mregs induced Treg differentiation and increased the release of Tregs in the local lymph node drainage in mice with nephrosis. In conclusion, it seems that there is extensive cooperation between MDSCs and other immunosuppressive cell populations in the regulation of inflammatory conditions.

### Increased myelopoiesis and expansion of MDSCs with aging

The inflammaging process is associated with significant changes in the hematopoietic system affecting the generation of myeloid and lymphoid cells in both humans and mice [98–100]. As the individual grows old, there is an increase in the rate of myelopoiesis, whereas lymphopoiesis clearly decreases in the bone marrow. This age-related imbalance in the immune system is caused by the myeloid-biased dominance of the hematopoietic stem cell (HSC) clones as compared to the progenitor clones of B and T lymphocytes. Moreover, a sizeable involution of the thymus with aging reduces lymphopoiesis, since thymus is an important lymphoid tissue in which T cells mature [101]. It is known that inflammatory mediators, e.g., CSFs, TNF-α, and interferons, originating from inflamed tissues can control myelopoiesis during the aging process [102, 103]. Inflammatory changes also appear with aging in the bone marrow which might also enhance aberrant myelopoiesis [104]. Currently, it is not known if it is the activation of MDSCs that controls the functions of HSCs and the progenitors of myeloid and lymphoid lineages in the bone marrow. However, there is substantial evidence that factors secreted by MDSCs, e.g., TGF-β, and IL-10, are potent regulators of HSCs [105, 106]. In particular, it has been claimed that aged HSCs were remarkably sensitive to TGF-β signaling. which might enhance myelopoietic differentiation [107]. Consequently, an age-related increase in myelopoiesis can enhance the production of myeloid cells, including MDSCs, and thus maintain inflammaging.

There is convincing evidence that the aging process increases the frequencies of circulating MDSCs, in both humans [108, 109] and mice [110]. Verschoor et al. [108] revealed that the levels of the CD11b^+^ CD15^+^-positive, granulocytic MDSCs were increased in the blood of community-dwelling seniors (61–76 years) and especially in frail elderly people (67–99 years). Recently, Alves et al. [109] demonstrated that the percentage of MDSCs was significantly higher in the blood of old people (80–100 years) than in their younger counterparts (20–30 years). Especially, the percentage of granulocytic MDSCs was robustly upregulated, while that of monocytic MDSCs was unaffected. It still needs to be clarified whether an increased myelopoiesis with aging in humans could increase the generation of MDSCs in the bone marrow, thus provoking the upregulation of MDSCs in the blood. However, there are several studies in mice revealing that the numbers of MDSCs are clearly increased with aging in the bone marrow, spleen, and peripheral lymph nodes [110–114]. The MDSCs isolated from the spleen of aged mice potently suppressed the antigen-induced T cell proliferation and T cell-dependent antibody production as well as inhibited the tumor cytotoxicity of T cells [110, 111]. The increased accumulation of MDSCs with aging was also linked to an enhanced growth of tumors in old mice. Flores et al. [114] reported that there were greater numbers of MDSCs in the bone marrow of two progeroid mouse species, i.e., *Ercc1* and *BubR1* mutants, than in wild-type mice. Currently, it is difficult to confirm whether the inflammaging process increases the level of MDSCs in peripheral tissues attributable to technical problems and the plasticity of MDSC phenotype.

Not only does the MDSC population of the immunosuppressive network expands with aging, but also the numbers of Tregs (CD25^+^ FOXP3^+^) increase in both elderly humans and mice [115–119]. This increase in the number of Tregs was significant in the spleen and lymph nodes, but also present in the skin. There were also age-related changes in the subtypes of Tregs, i.e., the number of naturally occurring thymus-derived Tregs (tTregs) increased with aging, whereas that of inducible Tregs (iTregs) seemed to decline in old mice [120]. Chougnet et al. [121] demonstrated that the aged Treg population was more resistant to apoptosis; this phenomenon was attributable to the reduced expression of pro-apoptotic Bim protein which might enhance the survival of aging Tregs. However, the Tregs from old mice were functionally active, i.e., they were able to prevent the activation of immune responses of effector T cells. Garg et al. [118] demonstrated that the Tregs from aged mice were more potent in inhibiting the proliferation of effector T cells than those isolated from young mice. Aged Tregs also secreted an increased level of the immunosuppressive IL-10 cytokine. Moreover, Garg et al. [118] presented evidences that the age-related increase in the expression of FOXP3^+^, the master regulator of Tregs, was induced by a hypomethylation of the enhancer sequences of *FoxP3* gene. Given that the interactions between MDSCs, Tregs, Bregs, and Mregs maintain the immunosuppressive milieu of tissues (Fig. 1), it is apparent that the age-related functions of Bregs and Mregs need to be clarified. There is an abundant literature on macrophage polarization with aging and in the repair process of tissue injuries [122, 123]. It seems that the responses are remarkably context dependent, probably attributable to the plasticity of macrophages and the complex regulation of the M1/M2 polarization process. Macrophage polarization can also fluctuate during the repair process [124]. However, Jackaman et al. [125] demonstrated that the numbers of anti-inflammatory M2 macrophages were robustly increased in the bone marrow, spleen, and lymph nodes of old mice as compared to their younger counterparts. Wang et al. [126] reported that the aging process in muscles was associated with an increase in the level of M2a macrophages, thus causing fibrosis in muscles. It is likely that the cooperation of tissue-resident macrophages with MDSCs and Tregs might switch these cells toward the immunosuppressive M2 phenotype during the aging process. For instance, MDSCs and Tregs secrete IL-10 and TGF-β, which polarize macrophages into the Mreg phenotype.

### Comparison of immune profiles of immunosenescence and MDSC-driven immunosuppression

Given that MDSCs are potent inducers of immunosuppression of adaptive immunity and a significant expansion of MDSCs and Tregs accompanies aging, this could induce and maintain a chronic state of immunosenescence. The MDSC-induced immunosuppression would represent the remodeling mechanism of immunosenescence. The remodeling of immune system might be crucial for the survival of tissues in conditions of chronic inflammation, e.g., in many pathological conditions and even in low-grade inflammaging. It is likely that MDSCs affect immune cells in a direct manner, but some responses detected in in vivo experiments can also be mediated via their interaction with other immunosuppressive cells, e.g., Tregs and Mregs (Fig. 1). Next, we will examine in more detail the similarities in the immune profiles generated by immunosenescence and the MDSC-induced immunosuppression in adaptive and innate immune systems.

### Adaptive immunity

#### T cells

There is an abundant literature indicating that immunosenescence is associated with a progressive decline in the numbers of naïve (CD45RA^+^) CD4^+^ and CD8^+^ T cells, whereas the numbers of the memory type (CD45RO^+^) of CD4^+^ and CD8^+^ T cells gradually increase with aging [7, 127–129] (Fig. 2). This hallmark of immunosenescence has been commonly observed in both humans and mice. The age-related loss of CD4^+^ and CD8^+^ T cells is attributable to a decline in the clonal expansion of T cell clones in the bone marrow and thymus, as well as to a decrease in their proliferation after maturation. In addition, there are aging-associated changes in the homeostasis of T helper (Th) cells and Tregs. The numbers of Tregs significantly increase with aging and, at the same time, there seems to occur reshaping in the numbers of Th populations. The production of Th1 and Th2 cytokines declines in elderly humans [130] and aging seems to be accompanied by a shift from the Th1 to the Th2 response, although results are more inconsistent in humans [131]. Interestingly, the presence of Th17 cells increases with aging in both humans and mice [132, 133]. Th17 cells are plastic cells which possess proinflammatory properties, e.g., they are involved in autoinflammatory diseases, but they can also exert antifungal and immunosuppressive effects, e.g., they can inhibit T cell-mediated immunity [134]. In addition to the changes in the subsets of T cells, there are significant age-related alterations in the functional capacities of both CD4^+^ and CD8^+^ T cells [135, 136]. In general, the responsiveness to distinct external insults decreases with aging in T cells, e.g., there are significant declines in both the proliferation and cytotoxicity of CD8^+^ T cells [137]. This loss of sensitivity is associated with clear age-related changes in the signaling responses of T cells which affect the differentiation of T cells and the immune outcomes of T cells in host defense [7, 138, 139].

TCRs and their co-receptors, e.g., CD28^+^, have an important role in the recognition of antigens and the activation of CD4^+^ and CD8^+^ T cells (Fig. 2). Recent high-throughput TCR sequencing studies have revealed that the structural TCR repertoire of naïve CD4^+^ and CD8^+^ T cell populations in humans dispersed with aging, indicating the non-uniform clonal proliferation of naïve T cells [140]. Moreover, Qi et al. [141] demonstrated that the diversity of the human TCR repertoire declined with aging in both naïve CD4^+^ and CD8^+^ T cells, although the clonal sizes of distinct phenotypes expanded with aging. Age-related changes were more modest in the memory CD4^+^ and CD8^+^ T cell populations. Several earlier studies have demonstrated that the aging process reduced the functional responses of TCRs [7], e.g., the formation of immune synapses with antigen presenting cells [142]. Aged human T cells also lose the expression and signaling of CD28 receptors which are crucial co-stimulators of TCR activation [143, 144].

T cells are the major target of MDSC-induced immune tolerance in tumors and several inflammatory disorders. MDSCs possess effective mechanisms to suppress the function of T cells and thus provide an immune escape not only for cancer cells, but also for organ allografts in transplantation medicine [145]. For instance, Nagaraj et al. [62] demonstrated that MDSCs were able to nitrate TCR proteins, inducing the dissociation of the TCR complex, which prevented the recognition of foreign antigens and thus suppressed the activation of T cells. Nitration might also affect the dispersion of the TCR repertoire with aging. Recently, Feng et al. [146] revealed that MDSC nitrated the lymphocyte-specific protein tyrosine kinase (LCK) and thus prevented the activation of TCR signaling. The inhibition of TCR signaling with nitration might induce T cell immunosuppression, not only in cancers and inflammatory conditions, but also in immunosenescence. In addition, MDSCs can suppress the functions of T cells by contacting them via the PD-1/PD-L1 checkpoint proteins. For instance, the activation of MDSCs clearly stimulated the expression of PD-L1 in human MDSCs [72]. Lu et al. [71] demonstrated that tumor-infiltrated MDSCs robustly expressed PD-L1 protein in human patients. In addition, Tregs induced the expression of PD-L1 (also called B7-H1) in MDSCs in mouse melanoma [147]. Although there is no direct evidence for the existence of these PD-L1-positive MDSCs in aged tissues, the microenvironment of inflammaging tissues contains different inflammatory factors which are activators of MDSCs [148]. Interestingly, Shimada et al. [149] reported that the expression of PD-1 was clearly increased with aging in mouse memory CD4^+^ T cells. Given that the PD-1/PD-L1 checkpoint system has an important role in the MDSC-induced immunosuppression, it seems likely that MDSCs could also exploit this mechanism to evoke T cell anergy and immunosenescence in inflammaging.

TGF-β is the major cytokine controlling the functions of the immunosuppressive network in a reciprocal manner. TGF-β is secreted by MDSCs, Tregs, and Bregs and has a crucial role in the proliferation and differentiation of T cells [150–152]. For instance, TGF-β inhibits the differentiation of Th1 and Th2 cells, whereas it enhances the differentiation of Tregs and Th17 cells. Interestingly, Th17 cells are very plastic cells which can possess pro-inflammatory properties, e.g., in autoinflammatory diseases, but they also exert immunosuppressive properties inhibiting T cell-based immunity. In addition, TGF-β was reported to reduce the cytotoxicity of CD8^+^ T cells, and the prevention of TGF-β signaling improved anti-tumor immunity [151]. Moreover, TGF-β enhanced the quiescence of hematopoietic stem cells, especially the development of lymphoid lineages declined with aging [153]. The TGF-β signaling maintains immune tolerance to both self and foreign antigens by controlling the differentiation and functions of effector T cells and Tregs. One could speculate that the increased presence of MDSCs and Tregs with aging might enhance the production of TGF-β and consequently augment the immunosenescence of T cells. The activation of MDSCs stimulates the expression of ARG1 and IDO which induces the metabolic catabolism of l-arginine and tryptophan. Some other myeloid cells can also express ARG1 and IDO proteins. In cancer studies, there is clear evidence that the induction of ARG1 and IDO in MDSCs stimulates T cell tolerance which enhances tumorigenesis [68, 70]. While it is known that aging affects arginine and tryptophan metabolism, it needs to be clarified whether immune effects are mediated by the depletion of these amino acids or through the production of their metabolites, i.e., NO from l-arginine and kynurenine metabolites from tryptophan [154, 155]. In conclusion, it seems that MDSCs induce the immunosuppressive profile of T cells which closely resembles the characteristics of the immunosenescence encountered in T cells.

#### B cells

B lymphocytes are generated in the bone marrow (BM) and subsequently immature B cells migrate into the spleen to undergo distinctive maturation and activation phases, e.g., the negative selection by self-antigens. In the aged human and mouse BM, there exists a myeloid-biased dominance of hematopoietic stem cell clones which downregulates the generation of lymphopoietic progenitors [100, 156]. The decline in the B cell progenitor clones also affects the development of the mature B cell compartment, another factor which enhances immunosenescence. The age-related increase in inflammatory changes in the BM, e.g., the increased presence of IL-1β and S100A9 factors, impairs B cell lymphopoiesis [19, 157]. Kennedy and Knight [158] demonstrated that MDSCs inhibited B lymphopoiesis through soluble factors in mouse BM cultures. Recently, they reported that inflammasomes might be involved, since exposure to the inflammasome inhibitor, glibenclamide, prevented the decline in B lymphopoiesis in the BM cultures [157]. Interestingly, they observed that the activation of inflammasomes promoted the development of MDSCs in the BM cultures. Flores et al. [114] revealed that the numbers of MDSCs were robustly increased in mouse BM with aging. The failure of B cell generation and the reductions in the numbers of these cells in the inflammatory BM may be caused by the TGF-β produced by MDSCs. It is known that TGF-β is a potent inhibitor of B cell proliferation and activation [47]. It can also enhance the apoptosis of immature and resting B cells. The numbers of human mature naïve B cells and the antigen-experienced memory (CD27^+^) B cells significantly decline with aging, whereas at the same time, the percentage of human late/exhausted memory B cells (CD27^−^) increase in the circulation [159, 160] (Fig. 2). Moreover, the diversity of the human B cell repertoire decreases with aging; this may reduce the responsiveness to infections and vaccination as well as increase the production of autoreactive antibodies [19, 156, 160, 161].

In addition to the inhibition of B cell development, MDSCs can also suppress the functions of mature human B cells, e.g., reduce their proliferation, homing, and antibody production [162–164]. MDSCs can inhibit the functions of B cells by secreting soluble factors, e.g., NO/ROS, PGE2, and TGF-β. Given that MDSCs can induce the expansion of Bregs and ameliorate autoimmunity [82], it seems that there exists a reciprocal regulation between MDSCs and Bregs, since the tumor-derived Bregs can educate both the monocytic and granulocytic MDSCs of mice and humans, by stimulating their immunosuppressive properties [165]. Moreover, Bregs can convert CD4^+^ T cells into Tregs [86] which are potent inhibitors of B cell functions. Therefore, it should be clarified whether immunosenescence affects the proliferation and functions of Bregs. However, it is known that certain inflammatory conditions increase the proliferation and immunosuppressive activities of Bregs [79]. It seems that chronic inflammation is the main cause of the functional impairments in the B cell compartment which accompany aging and thus it is likely that MDSCs are involved in the immunosenescence of B cells.

### Innate immunity

Currently, there is a debate about the role of innate immunity in the maintenance of immunosenescence. Clearly, age-related changes have been reported in the cells of innate immunity, but many observations are inconsistent, probably due to the fact that alterations are context dependent. The controversies may well be attributed to the high plasticity of myeloid-derived cells i.e., the cells of myeloid lineage can mature/convert into diverse myeloid subsets. For instance, MDSCs can differentiate into macrophages in inflammatory microenvironments (see above). Moreover, both a context-dependent polarization and a modulation of cell subsets are common characteristics of myeloid cells. The age-related changes in innate immunity have been described in detail elsewhere [166, 167]. We will focus on the modifications which have confirmed the potential associations with the MDSC-induced regulation.

#### Dendritic cells

One major function of dendritic cells (DCs) is the antigen processing and its presentation to T and B lymphocytes. Thus, DCs have a crucial role in the function of the adaptive immune system. It seems that the numbers of circulating DC subsets do not significantly change with aging, although there are conflicting results between studies. However, it appears that there is a decline in the numbers of Langerhans cells with aging in both human and mouse skin [168, 169]. It seems that aging affects several functions of DCs, although the results are not always consistent [20, 170]. There are both human and mouse studies indicating that the migration of DCs is impaired and their capacity to phagocytose antigens, process them, and subsequently present them to T cells decreases with aging [20, 170–172] (Fig. 2). However, contradictory observations have been reported. Moreover, Panda et al. [173] demonstrated that there was a significant age-related decrease in the amounts of cytokines induced by the activation of TLRs in human myeloid and plasmacytoid DCs. This may impair the priming of T cells and furthermore inhibit the polarization of Th cells. Currently, the mechanisms of cross talk between DCs and T cells need to be clarified, although a role for checkpoint inhibitors has been proposed [174]. In cancer studies, there is clear evidence that MDSCs inhibit the functions of DCs [78]. For instance, Greifenberg et al. [175] reported that the activation of MDSCs with LPS and IFN-γ prevented the differentiation of DCs in mouse BM cultures. Poschke et al. [176] demonstrated that the DCs which had been generated in a co-culture with human MDSCs displayed a reduced antigen uptake and impaired cytokine production. TGF-β1 is a potent inhibitor of the maturation and function of human and mouse DCs [56, 177]. However, there are differences between DC populations, since TGF-β1 was required for the development and maintenance of Langerhans cells in mice [178]. It seems reasonable to propose that MDSCs can inhibit the functions of DCs and, in this way, contribute to the suppression of T and B cells in cancers and inflammatory conditions.

#### Natural killer cells

Natural killer (NK) cells have an important role in innate immunity, although they are lymphocytes originating from lymphoid progenitors in the bone marrow. NK cells are cytotoxic cells and undertake similar functions as cytotoxic T cells in the host defense against cancer and viral infections [179]. There are diverse subsets of NK cells which have distinct functions mediated via their surface receptors; this is important not only in cytotoxicity, but also in the cross talk with other immune cells, e.g., DCs and T cells [180, 181]. It is known that aging affects the diversity of NK cell subsets, with this being reflected in the altered surface receptor phenotypes and expression levels, both in humans and mice [22, 182] (Fig. 2). These changes, which can be already detected in the bone marrow, lead to a decline in the cytotoxic capability of NK cells. In addition, the ability of human NK cells to produce cytokines and chemokines significantly decreases with aging. The impaired cytotoxicity of the NK cell population exposes elderly people to tumorigenesis and viral and bacterial infections. There is convincing evidence that MDSCs can inhibit the secretion of cytokines and reduce the cytotoxic properties of NK cells. For instance, Hoechst et al. [183] demonstrated that human MDSCs isolated from hepatocellular carcinoma (HCC) robustly inhibited the cytotoxicity of NK cells from HCC patients. The suppression of NK cells was independent of ARG1 and iNOS expression, but highly dependent on cell contacts which were mediated through the NKp30 receptors present on the NK cells. Infection studies with mice have revealed that granulocytic MDSCs inhibited both the proliferation and the activity of NK cells in response to adenovirus and vaccinia virus infections [184, 185]. There seems to be different mechanisms mediating the inhibition of NK cells evoked by MDSCs. For instance, MDSCs can inhibit the activity of NK cells by secreting ROS [184] and NO [186] or suppress the functions of NK cells via the membrane-bound TGF-β signaling [187]. It is known that TGF-β is a potent inhibitor of the development and differentiation of human NK subsets [188]. These observations indicate that MDSCs could induce the kinds of changes observed in the functions of NK cells with aging.

#### Monocytes and macrophages

There are many cell types participating in the innate immune system e.g., monocytes, macrophages, and granulocytes; neutrophils make up the largest group of granulocytes. There are less consistent results on the effects of aging on the functions of these cell types, since their functions tend to fluctuate with respect to the phase and intensity of inflammation. For instance, monocytes can differentiate into tissue macrophages in conditions of acute inflammation and macrophages can display M1/M2 polarization. However, there are several studies indicating that there are impairments in the properties of macrophages with aging, e.g., lowering of chemotaxis, antigen presentation, and phagocytosis [21, 123] (Fig. 2). The Toll-like receptor (TLR) signaling and its responses are impaired with aging in both humans and mice [189, 190]. It seems that the age-related alterations in macrophages are dependent on the tissue microenvironment and the disease-associated pathology, especially with respect to macrophage polarization [123]. For instance, tumors contain specific immunosuppressive TAMs which have both overlapping and distinguishing properties as compared to M2 macrophages [191]. Currently, it is not known whether there are specific age-associated M2 macrophages. MDSCs and macrophages have an important role in the resolution of inflammatory reactions [37, 40, 192]. Since MDSCs secrete both IL-10 and TGF-β, they can suppress proinflammatory functions and trigger the resolution phase, e.g., by inducing the M2 polarization of macrophages. Given that inflammaging evokes both inflammatory and anti-inflammatory responses, it seems that MDSCs suppress adaptive immunity and control innate immunity in a context-dependent manner.

#### Immunosenescence and MDSC-driven immunosuppression in inflammatory disorders

The hallmarks of immune system senescence not only appear with the aging process, but also evidences of premature immunosenescence are present in chronic inflammatory diseases. Sepsis has turned out to be an important model for elucidating the interactions between inflammation-induced immunosuppression and immunosenescence [193, 194]. Sepsis stimulates emergency myelopoiesis which induces the expansion of MDSCs [36, 194]. Consequently, these MDSCs induce a profound immunosuppression which is comparable to that present in cancer or age-related immunosenescence. However, it seems that MDSCs have a complex, phase-dependent role in the pathology of sepsis, causing tissue repair or its destruction. Brudecki et al. [195] utilized the mouse polymicrobial sepsis model to demonstrate that during the early phase, MDSCs secreted NO and pro-inflammatory cytokines, whereas in the later chronic phase, MDSCs expressed ARG1, IL-10, and TGF-β proteins. This indicates that within the course of sepsis, the properties of MDSCs shift from a proinflammatory phenotype to one with a strongly immunosuppressive character. Autocrine and paracrine immune factors induce the generation of immunosuppressive MDSCs in conjunction with Tregs and M2 macrophages, which in cooperation facilitate the resolution of infection. Moreover, the activation of ARG1 produces ornithine and polyamines which enhance the repair process. It has been reported that MDSCs induce immune suppression and also augment repair processes after a spinal cord injury [196] and acute kidney injury [197].

Autoimmune diseases display the hallmarks of premature immunosenescence [198]. Currently, there is convincing evidence that MDSCs have a crucial role in several autoimmune diseases, e.g., multiple sclerosis, rheumatoid arthritis, psoriasis, and autoimmune encephalomyelitis [46, 199–201]. In fact, many different autoimmune diseases are associated with an increase in the numbers of MDSCs in the spleen and lymph nodes, as well as in the tissues suffering autoimmune pathology and, moreover, these alterations correlate with the extent of the damage. Iacobaeus et al. [202] demonstrated that there appeared to be clear changes in the numbers of MDSCs between the relapse and remission phases in multiple sclerosis patients. The numbers of both monocytic and granulocytic MDSCs significantly increased during the relapse phase as compared to the stable phase. Experiments conducted in mice have revealed that MDSCs have a protective role against multiple sclerosis [199, 203, 204]. Similar observations have been found in autoimmune arthritis [205, 206]. Fujii et al. [205] demonstrated that collagen-induced arthritis (CIA) in mice robustly increased the numbers of MDSCs in the spleen. Splenic MDSCs effectively suppressed the proliferation of CD4^+^ T cells and inhibited their differentiation into Th17 cells, the major inducers of arthritic inflammation. They also revealed that the adoptive transfer of MDSCs alleviated the severity of CIA. However, there are contrasting observations indicating that MDSCs might promote the polarization of Th17 and thus augment mouse arthritis and encephalomyelitis during long-term exposures [207, 208]. Moreover, Wang et al. [209] observed that changes could occur in the type of MDSCs and their immunosuppressive properties during the course of disease which might impair efficient immunosuppression. The studies on sepsis and autoimmune diseases have clearly indicated that MDSCs induce immunosuppression (i.e., immunosenescence) which reduces the level of inflammation and the severity of injuries in inflamed tissues. It still needs to be clarified whether the MDSC-induced immunosenescence is also a feasible remodeling mechanism against inflammaging.

### Immunosenescence: cellular senescence of immune cells or inflammation-induced remodeling of the immune system?

The primary cause of immunosenescence is still uncertain, although the age-related senescence of the immune system was discovered more than four decades ago. Immunosenescence seems to have a multifaceted origin, since the aging process affects the development and maturation processes of immune cells, e.g., via thymic involution, as well as their functions in peripheral, mildly inflamed tissues (Sects. 4 and 5). Given that the proliferation of T and B cells declines with aging, this implies that immune cells could undergo cellular senescence, in the same way as non-immune cells. Several research groups have investigated the replicative senescence of T cells, both in in vivo and in vitro conditions. There is evidence that changes in surface markers of T cells, e.g., lack of CD28 expression, might cause an attrition of telomeres [210, 211]. However, it seems that the markers of cellular senescence are not identical in fibroblasts and immune cells, although, for instance, CD8^+^ T and memory B cells can express the senescence-associated secretory phenotype (SASP), a common cellular marker of non-immune senescence [212, 213]. Recently, Ong et al. [214] identified a non-classical monocyte subset in elderly people which displayed a pro-inflammatory SASP phenotype as well as many other hallmarks of cellular senescence. Vicente et al. [215] have reviewed the role of cellular senescence in the control of cell fate and functions of many immune cells. In this respect, we need to take into consideration the difference between quiescence and senescence, since many immune cells, e.g., naïve T cells, are in a quiescent state displaying cell cycle arrest and hyporesponsiveness although they are not senescent [216]. For instance, Tregs can induce and maintain the quiescence of memory CD8^+^ T cells [217]. In view of the continuous production of immune cells, it seems likely that immune cells are not truly irreversibly senescent but rather exhausted, exhibiting reduced functional capabilities [218].

As long ago as 1978, Roder et al. [219] made the interesting observation that mouse immunological senescence was associated with an increased activity of suppressor cells, especially in the spleen and bone marrow. They reported that suppressor cells secreted soluble mediators, which affected the characteristics of T cells and macrophages. Remarkably, the antibody responses of immune cells could be restored by specifically activated T cells and LPS, which indicated that immunosenescence was not caused by the lack of competent immune cells. After this seminal observation, a network of immunosuppressive cells has been identified. It seems that TGF-β, IL-10, and NO, secreted by MDSCs, are the major soluble mediators maintaining the functions of this age-related immunosuppressive network. There is an abundant literature indicating that TGF-β signaling suppresses the functions of CD4^+^ [220] and CD8^+^ [221] T cells as well as DCs [222] and NK cells [223]. In particular, TGF-β inhibits the signaling pathways of CD28 and mTOR kinase. IL-10 also inhibits the CD28-mediated signaling in T cells by activating SHP-1 tyrosine phosphatase-1 [224]. TGF-β also has an important role in the functions of HSCs, e.g., TGF-β signaling promotes the myeloid differentiation of distinct mouse HSC subtypes, thus stimulating myelopoiesis with aging [225]. Flavell et al. [151] have reviewed the immune-suppressive effects of TGF-β on cells in both the innate and adaptive immune systems. Interestingly, many TGF-β-induced responses are the same as those observed in immunosenescence. IL-10, a cytokine produced by MDSCs, Tregs, and Th2 cells, also possesses different immunosuppressive functions and maintains the homeostasis of host tissues [48]. It seems that the MDSC-driven immunosuppressive network is able to generate the phenotypes in the cells of adaptive and innate immunity which are comparable to those appearing in immunosenescence (Fig. 2), although direct evidence on the causal role of MDSCs needs to be clarified.

### Outlines for future studies

Currently, the causal role of MDSCs and other immunosuppressive cells in the generation of immunosenescence needs to be clarified, although there is a clear similarity between the immune cell phenotypes induced by either MDSCs or the aging process involving a low-grade inflammation. The studies on cancer therapies have revealed surprisingly many chemotherapeutic and immunotherapeutic treatments which suppress the functions of MDSCs [226–228]. There are different therapeutic strategies which target e.g., (1) the maturation process of MDSCs, (2) the trafficking of MDSCs into tumors, (3) the expansion and activation of MDSCs, and (4) the depletion of MDSCs. For instance, distinct compounds, e.g., all-trans retinoic acid (ATRA) and β-glucan, can induce the maturation of MDSCs into the cells of innate immunity [229, 230]. In addition, the inhibitors of signaling pathways, e.g., the inhibitors of STAT3 and COX-2/PGE2, can reduce the activation of MDSCs [228]. Several phytochemicals are also able to inhibit the function of MDSC and thus can alleviate immunosuppression in tumors and inflammatory diseases [231]. However, chemotherapeutic compounds do not specifically target MDSCs and thus there is intense search for the specific antigens of MDSCs which could be targeted in immunotherapies. Recently, Dominguez et al. [232] reported promising results that the agonistic TRAIL-R2 antibody selectively eliminated MDSCs without affecting other immune cells. It is important to understand whether the inhibition of MDSCs function in aged mammals could reverse immunosenescence and thus provide insight into the origin of immunosenescence. As far as we know, this approach has not been utilized in studies attempting to find ways to rejuvenate the immune system of elderly people or primates [233, 234]. Inhibiting the functions of MDSCs and other immunosuppressive regulators could provide the target to reverse the process of immunosenescence (i.e., induce rejuvenation), using the same approach which improves the immune surveillance of tumors and infections. This might also clarify the observations that the aging process increases the risk for cancers and chronic infections. There are studies on the combination therapies indicating that blocking the function of MDSCs, e.g., by ATRA and entinostat, improved immunotherapies in cancers and antibiotic treatments in infections [235–237].

There are many studies on tumors where the phenotypes of MDSCs and other immune cells have been identified, whereas in immunosenescence the phenotypes of immune cells in non-immune tissues have not been characterized. Especially, an interesting question is whether there is an accumulation of MDSCs and other immunosuppressive cells in aging tissues in association with an increased level of markers of chronic low-grade inflammation. It is known that the presence of MDSCs remarkably increases with aging in the bone marrow, spleen, and lymph nodes, but no studies exist on peripheral tissues. The great plasticity of MDSCs might cause problems, since MDSCs are disposed to mature toward M2 macrophages in inflamed tissues (see above). Technical problems might also appear with non-immune tissues, since cell sorting techniques are required for the analysis of MDSCs. However, the presence of MDSCs has been verified in studies on age-related diseases in different tissues. This approach will exclude the possibility that the age-related increase in the level of MDSCs in the blood and immune organs could be caused by age-related pathologies, such as tumors. The appearance of other cooperative partners of immunosuppression, i.e., Tregs, Mregs, and Bregs, should also be analyzed at the tissue level, since their immunosuppressive armament not only affects immune cells, but also induces harmful bystander effects on neighboring host tissue [148].

## Conclusions

The role of immunosenescence in the aging process still needs to be clarified. There is an extensive literature related to the age-related decline in the function of immune system, but it has proved difficult to determine whether the overall effects are beneficial or detrimental. Given that the perpetrator inducing the age-related mild inflammatory profile cannot be eliminated, immunosenescence seems to be an important remodeling mechanism attempting to maintain tissue homeostasis as the individual grows old. Although it has been known for four decades that the aging process is associated with a suppression of immune system, the mechanism behind this phenomenon has remained elusive. Currently, it is known that there exists a network of immunosuppressive cells which exploit a wide spectrum of mechanisms to inhibit the excessive functions of the immune system. There is abundant evidence indicating that MDSCs are potent immunosuppressive cells in diverse inflammatory conditions, especially in tumor-associated inflammation. MDSCs also co-operate with other immunosuppressive cells, e.g., Tregs, Bregs, and Mregs, to suppress immune functions in inflammatory conditions. Interestingly, the numbers of MDSCs increase with aging which supports the proposal that they have a crucial role in the coordination of immunosenescence.
